# Case Report: Sustained Efficacy of Lumasiran at 18 Months in Primary Hyperoxaluria Type 1

**DOI:** 10.3389/fped.2021.791616

**Published:** 2022-01-05

**Authors:** Benedetta Chiodini, Nathalie Tram, Brigitte Adams, Elise Hennaut, Ksenija Lolin, Khalid Ismaili

**Affiliations:** Department of Pediatric Nephrology, Hôpital Universitaire des Enfants Reine Fabiola, Université Libre de Bruxelles, Brussels, Belgium

**Keywords:** primary hyperoxaluria, lumasiran, RNAi therapeutic, kidney stones, children

## Abstract

**Background:** Primary hyperoxaluria type 1 (PH1) is a rare genetic disease caused by hepatic overproduction of oxalate, ultimately responsible for kidney stones, kidney failure and systemic oxalosis. Lumasiran, is a liver-directed RNA interference therapeutic agent. It has been shown to reduce hepatic oxalate production by targeting glycolate oxidase, and to dramatically reduce oxalate excretion.

**Care Report:** We present the case of a teenager patient affected by PH1, who entered in the lumasiran compassionate use program. The patient had a rapid and sustained decrease in urinary oxalate/creatinine ratio, with a mean reduction after lumasiran administration of about 70%. During the 18 months long follow-up, urinary oxalate remained low, reaching nearly normal values. Plasma oxalate also decreased dramatically. Normal levels were reached immediately after the first dose and remained consistently low thereafter. During the same follow-up period, eGFR remained stable at about 60 ml/min/1.73 m^2^, but no new kidney stones were observed. Existing kidney stones did not increase in size. The patient did not suffer renal colic events and did not require further urological interventions.

**Conclusion:** In our severely affected PH1 patient, lumasiran proved to be very effective in rapidly and consistently reducing plasma oxalate and urinary excretion to normal and near-normal levels, respectively. In the 18 months long follow-up post-lumasiran, the eGFR remained stable and the patient showed clinical improvements. As far as we know, this report covers the longest observation period after initiation of this novel RNAi therapy.

## Introduction

Primary hyperoxaluria type 1 (PH1) is a rare autosomal recessive disease caused by an enzymatic defect of the alanine-glyoxylate amino-transferase (AGT). This deficiency results in the overproduction of oxalate which is mostly excreted by the kidneys in the form of calcium oxalate crystals. Oxalate deposition causes recurrent nephrolithiasis, nephrocalcinosis and may eventually damage the tubules, leading progressively to a loss in kidney function and systemic oxalosis (1, 2).

The clinical expression of PH1 ranges from occasional or recurrent urinary lithiasis in adults and teenagers to the most severe spectrum with early end-stage kidney disease (ESKD) developing already in infancy (3). As urinary oxalate levels are known to be a principal determinant for the progression toward ESKD (4), the management of PH1 is primarily directed at reducing urinary oxalate levels. However up to now, the treatment of PH1, consisting in hyperhydration, high-dose pyridoxine and calcium oxalate crystallization inhibitors as citrate, has often proven insufficient. In severe cases, once ESKD is reached, liver transplantation (followed or combined with kidney transplantation) has been the only therapeutic option able to treat the metabolic defect and normalize oxalate levels. This was true until today.

New therapeutic options are now foreseeable. Lumasiran, a subcutaneously administered, liver-directed RNA interference (RNAi) therapeutic agent, has been shown to reduce hepatic oxalate production by targeting glycolate oxidase. In a phase 1-2 study, lumasiran had an acceptable safety profile and reduced 24-h urinary oxalate excretion in all patients with PH1 to near-normal levels ( ≤ 1.5 times the upper limit of normal) (5). In 2019 and for 6 months, the double-blind phase 3 clinical trial ILLUMINATE-A evaluated the efficacy and safety of lumasiran in 39 patients with PH1 older than 6 years of age with no ESRD (eGFR of at least 30 ml per min per 1.73 m^2^ of body surface area) and no systemic oxalosis (6). Lumasiran reduced urinary oxalate excretion and most of the patients reached normal or near-normal levels after 6 months of treatment (6).

## Case Report

M.S. presented for the first time to a tertiary hospital in Brussels in October 2015 at the age of 9 years because of intense abdominal and lumbar pain. Originating from a consanguineous family from Iraq, the boy and his parents had only recently moved to Belgium. Since the age of 3 years, M.S. had been treated for stones in Iraq by extracorporeal lithotripsy and percutaneous nephrolithotomy, although the family was unaware of the precise diagnosis.

At entry, a first ultrasound and tomographic scan showed multiple bilateral coralliform stones. A large stone was trapped in the left pelvis. The biological analyses revealed a mildly increased plasma creatinine (creat 0.75 mg/dL) and an excessively high urinary oxalate/creatinine ratio (0.21 mol/mol; normal values <0.06 mol/mol).

At the genetic investigation, the patient resulted homozygote for a duplication of the entire exon 9 on the AGXT gene, confirming the diagnosis of PH1.

The patient was started on hyperhydration (>2 L/m^2^ per day) and potassium citrate as crystallization inhibitor (100–150 mg/Kg per day). As dietary regimen, a limited salt intake was recommended. A trial with high-dose pyridoxine was stopped 3 months after the treatment initiation, as no decrease in the level of urinary oxalate was observed.

In terms of surgery, between 2015 and 2020, the child underwent an average of 5 urological interventions per year, including placement and withdrawal of ureteral double J stents, ureteroscopy, laser therapy for stone extraction and extracorporeal lithotripsy.

During this period, the eGFR and urinary oxalate/creatinine ratio remained stable (average of 67 ml/min/1.73 m^2^ and 0.28 mol/mol, respectively), while the plasma oxalate increased from 20 micromol/L at presentation to 32 micromol/L 4 years later (normal values <27 micromol/L). Although no deposition of calcium oxalate crystals in other tissues was observed, the formation of new kidney stones was constant, albeit the medical treatment and the numerous and aggressive surgical interventions.

In March 2020 at the age of 13, M.S. entered in the lumasiran compassionate use program (NCT04125472). At that time, the ILLUMINATE-A trial was ongoing, but the inclusion period was over (6).

**Figure 1:**
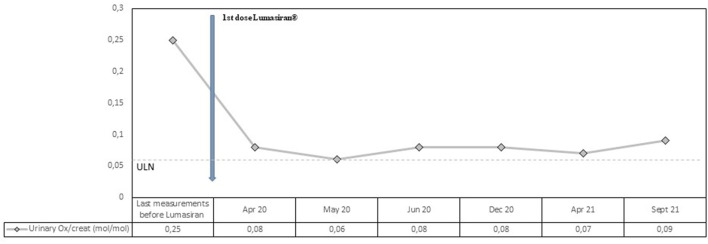
Observed values for Urinary Oxalate/creatinine ratio from the last measurement before lumasiran to Month 18. ULN denotes upper limit of the normal range.

At the end of March 2020, M.S. received the first injection of lumasiran subcutaneously at the dose of 3 mg per kilogram of body weight. As in the ILLUMINATE-A design, the first three doses have been administered once per month, followed by maintenance doses given once every 3 months beginning 1 month after the last loading dose (5). The patient showed a rapid and sustained decrease in urinary oxalate/creatinine ratio (see Figure 1). The decline was seen immediately after 1 month (from 0.25 to 0.08 mol/mol; normal values <0.06 mol/mol) with a mean reduction in urinary oxalate/creatinine ratio after lumasiran administration of about 70%. During the 18 months follow-up period, urinary oxalate remained low reaching nearly normal values (see Figure 1).

**Figure 2:**
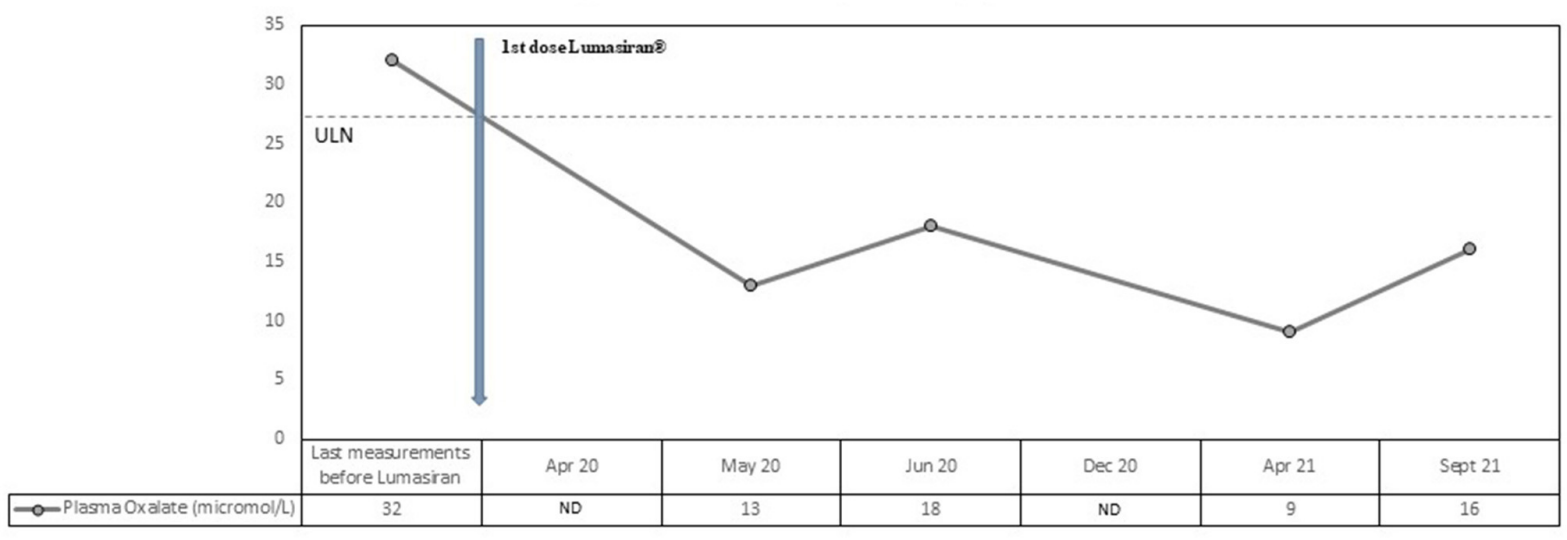
Observed values for Plasma Oxalate from the last measurement before lumasiran to Month 18. ULN denotes upper limit of the normal range.

Plasma oxalate levels also decreased rapidly and dramatically after the first lumasiran injection (from 32 micromol/L to 13 micromol/L; normal values <27 micromol/L). Normal plasma oxalate levels were reached immediately after the first dose and the values remained consistently low during the 18 months follow-up period (see Figure 2). The mean reduction in plasma oxalate level after lumasiran administration was about 60%.

The eGFR remained stable between the pre- and post-administration period, ranging from 60 ml/min/1.73m^2^ at baseline to 62 ml/min/1.73m^2^ 18 months later.

Kidney ultrasonography, which was performed at baseline and at the time of each lumasiran injection, showed no substantial changes. However, in the 18 months follow-up post-lumasiran, no new stones were observed, and the existing ones did not increase in size. The patient did not suffer renal colic events and did not require urgent urological intervention.

In terms of safety, no severe or serious adverse event was observed, apart from temporary and asymptomatic episodes of macroscopic hematuria after the first four Lumasiran injections.

## Discussion

Oxalate is the toxic metabolite responsible for kidney stones, ESKD and systemic oxalosis in PH1. Reduction in hepatic oxalate production, measured with the use of plasma and urinary oxalate, is the only effective treatment for this disease. Up to now, liver transplantation was the only available option to achieve this goal. Lumasiran, the new liver-directed RNAi therapeutic agent, opens new prospects. By silencing the gene encoding glycolate oxidase, lumasiran depletes glycolate oxidase, inhibiting the synthesis of oxalate. In the recent ILLUMINATE-A trial, the 24 patients assigned to the lumasiran group showed a decrease in the 24-h urinary oxalate excretion level of nearly two-thirds, with an effect seen already 1 month after the first dose (6). After 6 months, 84% of those patients reached a 24-h urinary oxalate excretion level no higher than 1.5 times the upper limit of the normal range (6). The observed percent reduction in urinary oxalate/creatinine ratio in spot urinary sample was comparable to the decrease in the 24-h urinary oxalate excretion, adding to existing data that supports the use of spot urine samples as an acceptably accurate and simple method to measure changes in urinary oxalate excretion (6, 7). While in the 6 months ILLUMINATE-A trial, lumasiran also proved effective at reducing plasma oxalate levels significantly (6), no real change could be seen in terms of eGFR. It is possible that the improvement of nephrocalcinosis (and eGFR consequently) will need a much longer period span to be perceived, as it is often observed after liver transplantation (8).

Our patient was affected by a severe form of PH1 started lumasiran at the age of 13 and since then has been followed for 18 months. Consistently with the ILLUMINATE-A trial, he showed a rapid and sustained decrease in urinary oxalate/creatinine ratio, with a mean reduction after lumasiran administration of about 70%. From the first month post-lumasiran and during the 18 months follow-up, urinary oxalate remained low, reaching nearly normal values (see Figure 1). Plasma oxalate level also decreased rapidly and dramatically after the first lumasiran injection, reaching normal levels already after the first dose (see Figure 1). The eGFR and the sonographic images remained stable. However, during the 18 months follow-up post-lumasiran, no new kidney stones were observed, and the existing kidney stones did not increase in size. The patient did not suffer renal colic events and did not require further urological interventions.

In terms of safety, no severe or serious adverse event was observed. The only events reported after the first four lumasiran injections were temporary episodes of macroscopic hematuria comparable to the episodes experienced before treatment initiation.

In conclusion, in our severely affected PH1 patient, lumasiran proved to be very effective in rapidly and consistently reducing plasma oxalate and urinary excretion to normal and near-normal levels. During the 18 months follow-up post-lumasiran, no deterioration in the kidney images and eGFR has been observed. This can be considered a satisfying result when compared to the natural evolution of the disease with conservative treatment. Moreover, during this period the patient did not produce new stones and remained free of renal colic events and urgent urological interventions. Though our paper has the main limitation of being a single case report, as far as we know, it covers the longest observation period after initiation of this novel RNAi therapy.

## Data Availability Statement

The original contributions presented in the study are included in the article/supplementary material, further inquiries can be directed to the corresponding author.

## Ethics Statement

The treatment was approved by the Local Institutional Review Board (CEH n°43/20).

## Author Contributions

BC: drafting the manuscript, revising the manuscript, and final approval of the version to be published. NT: building Figure 1, revising the manuscript, and final approval of the version to be published. BA, EH, KL, and KI: revising the manuscript and final approval of the version to be published. All authors contributed to the article and approved the submitted version.

## Conflict of Interest

In 2020, BC and BA participated in the Advisory Board for Alnylam. The remaining authors declare that the research was conducted in the absence of any commercial or financial relationships that could be construed as a potential conflict of interest.

## Publisher's Note

All claims expressed in this article are solely those of the authors and do not necessarily represent those of their affiliated organizations, or those of the publisher, the editors and the reviewers. Any product that may be evaluated in this article, or claim that may be made by its manufacturer, is not guaranteed or endorsed by the publisher.
